# The Importance of Monitoring Cortisol in the Agri-Food Sector—A Systematic Review

**DOI:** 10.3390/metabo13060692

**Published:** 2023-05-26

**Authors:** Dayana Aguiar, Carlos Marques, Ana C. Pereira

**Affiliations:** 1 Department of Physics, University of Aveiro, 3810-193 Aveiro, Portugal; dayana.aguiar@staff.uma.pt (D.A.); carlos.marques@ua.pt (C.M.); 2 ISOPlexis, Centre for Sustainable Agriculture and Food Technology, University of Madeira, Campus da Penteada, 9020-105 Funchal, Portugal; 3 Institute of Nanostructures, Nanomodelling and Nanofabrication (I3N), University of Aveiro, 3810-193 Aveiro, Portugal; 4 Chemical Process Engineering and Forest Products Research Centre, Department of Chemical Engineering, University of Coimbra, Pólo II—Rua Sílvio Lima, 3030-790 Coimbra, Portugal

**Keywords:** stress monitoring, cortisol, animal welfare, food industry, extraction, LC-MS/MS

## Abstract

Cortisol monitoring in the agri-food sector is considered a valuable tool due to its direct correlation with growth, reproduction, the immune system, and overall animal welfare. Strategies to monitor this stress hormone and its correlation to food quality and security have been studied in fish farming and the livestock industry. This review discusses studies on monitoring cortisol in the food industry for the first time. The impact of cortisol on animal production, quality, and the security of food products, and the analytical procedures commonly implemented for sample pre-concentration and quantification by liquid chromatography coupled to mass spectrometry, are reviewed and discussed according to the results published in the period 2012–2022. Aquaculture, or fish farming, is the leading agri-food sector, where cortisol’s impact and usefulness are better known than in livestock. The determination of cortisol in fish not only allows for an increase in the production rate, but also the ability to monitor the water quality, enhancing the sustainable development of this industry. In cattle, further studies are needed since it has mainly been used to detect the administration of illicit substances. Current analytical control and monitoring techniques are expensive and often depend on invasive sampling, not allowing fast or real-time monitoring.

## 1. Introduction

Over the last few years, the agri-food sector has adopted sustainable and innovative approaches to guarantee and improve food safety, product quality and respect for the environment, and to minimize or avoid food waste [1].

In this context, animal welfare is essential to ensure human food quality and accelerate the development of agri-food systems. Animal well-being is easily affected by environmental alterations that induce behavioral and physical changes associated with stress and help them cope with their environment and adapt to challenging situations rapidly. On the other hand, they can impair critical biological functions. This response emerges via the activation of the hypothalamic–pituitary–adrenal axis (HPA axis), which produces steroid hormones. One of the most important hormones produced is cortisol, a lipid-soluble steroid hormone that can diffuse through cell membranes and is not stored. This glucocorticoid is realized when the HPA axis is activated by a stimulus such as environmental changes, capture, handling, contaminants or pollutants, predators, and transportation, causing stress. The effect of cortisol on the living organism will be influenced by the abundance of type I and type II glucocorticoid receptors and the levels of free glucocorticoid that are within the tissue and able to bind to this receptor [2]. The stress response usually emerges to promote an adaptative reaction by activating the glucose metabolism. Still, when it is repeatedly or continuously activated over long periods, it can be deleterious to health. This hormone has glucoregulatory actions, influencing the genes that control essential functions of living organisms, such as metabolism, growth, repair, and reproduction. However, when cortisol is sustained at high levels, it can promote immunosuppression, reproduction suppression, and tissue atrophy. According to the review of Sadoul and Geffroy [3], cortisol can be metabolized and inactivated in fish, principally through the hepato-biliary–fecal route. In the liver and the bile, the cortisol is inactivated by reduction and conjugation to glucuronide or sulfate. Supplementary information regarding a detailed mechanism description of cortisol release and inhibition can be found in Ralph et al. [2] and Leatherland et al. [4]. Cortisol is the primary corticosteroid produced by mammals and fishes, whereas it is corticosterone for reptiles and birds [2,5,6].

Monitoring cortisol in animal studies is commonly used to identify stress responses and welfare threats. This stress hormone can be found in several matrices of living organisms. Typically, cortisol is measured in blood plasma, tissue, or whole-body extracts, being considered an invasive process due to the inducement of stress caused by handling and, consequently, the production of the hormone within minutes that could easily bias the basal cortisol estimations. Non-invasive methods have emerged through detecting cortisol on other matrices, such as urine, feces, and saliva, to avoid that situation [7,8]. Despite being non-invasive for the species, accurately measuring cortisol requires rapid sample collection to prevent steroid degradation or contamination due to cortisol in the water, food debris or plant hormones, and the cortisol excretion in biological samples such as feces can be affected by the gut microbiota or by bacteria in the urine. Moreover, several freeze–thaw cycles can alter the hormones’ concentration [3,8].

In the agri-food sector, cortisol monitoring has been performed in several animal species since it is an excellent approach to assess animal welfare and allows the detection of alterations in their surrounding habitat or the application of illicit substances during their development. In fish, cortisol can be accurately and reliably measured in several matrices (i.e., plasma, whole body, eggs, urine, fins, scales, and mucous). However, it is not frequently measured during fish farming. Although the sampling method in most cases is invasive, monitoring this hormone is extremely important to ensure a reasonable growth rate at several stages of fish production and assess egg quality. The challenge is finding non-invasive alternatives to measure cortisol, for instance, in water [3,9,10]. According to Sadoul and Geffroy [3], measuring cortisol in water is useful. Once released into the water, cortisol has a half-life of 16 h at 12 °C, and accurate measurements can be achieved since the hormone accumulates readily in the water [3]. In contrast, investigations of cortisol in livestock animals are still emerging in the sense of its presence in animal products such as milk or the administration of prohibited substances that significantly increase weight or mask diseases before the animal’s sale [11,12,13].

The quantification of cortisol, in general, is only achieved after submitting the biological sample to an extraction or pre-concentration step, since it is routinely found in very low concentrations, on the order of ppt (ng/L) or ppb (µg/L). The most common techniques are liquid–liquid extraction (LLE) and solid–phase extraction (SPE), which are time-consuming and expensive. The most reported solvents used are ethyl acetate and methyl tertiary-butyl ether (MTBE). Nowadays, cortisol measurements are conducted using liquid chromatography coupled with tandem mass spectrometry (LC-MS/MS) due to its sensitivity, accuracy, automation, and the simultaneous detection of several steroid hormones in the same analysis [13,14,15]. However, this analytical technique requires an investment in instruments and maintenance, and the necessary internal standards are quite expensive.

Additionally, methods based on cortisol antibodies or immunoassays such as radioimmunoassay (RIA) and enzyme-linked immunosorbent assay (ELISA) have been developed. However, the measurements performed with immunoassays can be influenced by constituents present in the sample, and they are sensitive to incubation time and temperature [3]. The continuous development of the agri-food sector demands the development of a novel technique with low invasiveness, low cost, fast or real-time measurements, and reasonable specificity, and repeatability without waste production.

This review aims to provide an overview of cortisol’s impact in the agri-food sector, highlighting the matrices from which it is measured. We also summarize the analytical methods used for sample preparation to quantify cortisol in food samples. The focus is on the studies published in the last 11 years, and the systematic review was conducted according to the PRISMA methodology. To our knowledge, this is the first review published on this topic.

## 2. Materials and Methods

### 2.1. Information Sources and Search Strategy

**Figure 1:**
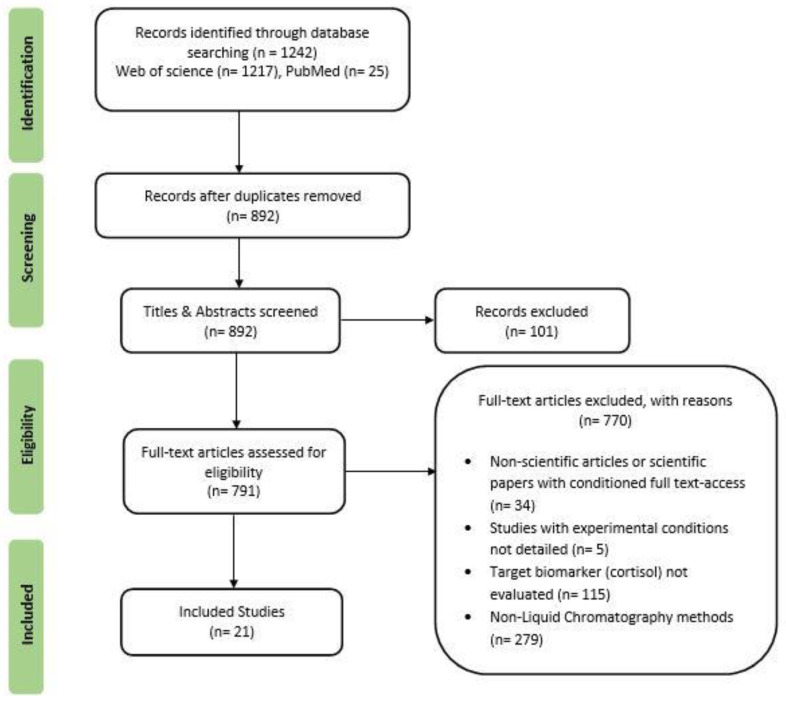
PRISMA flowchart of studies included in the current review.

A systematic review was carried out according to the PRISMA 2015 guidelines to consolidate the results currently available regarding cortisol’s impact on the agri-food sector and its detection and measurement [16]. The literature search was performed on two electronic databases, MEDLINE (PubMed) and Web of Science, and included studies published from 2012 to 24 June 2022 (date last searched). The search string included the term “Cortisol” together with “Detection methods” or “Quantification methods” or “Extraction methods” or “Non-invasive detection” or “Water detection” or “Aquaculture detection”. These terms were matched to the titles, abstracts, and keywords of articles written in English between 2012 and 2022. Figure 1 depicts the process utilized for collecting, selecting, and summarizing the currently available data regarding the importance and measurement of cortisol in food industry samples.

### 2.2. Eligibility Criteria

The two databases retrieved a total of 1242 records. This list of publications was first screened based on the title, authors, and year. Duplicate articles were removed. Next, the titles and abstracts were screened, excluding review studies, books or book sections, surveys, and conference proceedings. The present review article included and analyzed only research papers.

The “Eligibility” of the articles was determined according to the following five exclusion criteria: (i) full-text not available; (ii) studies with experimental conditions not detailed; (iii) target biomarker (cortisol) not determined; (iv) research papers that determined cortisol through non-liquid chromatography methods; and (v) studies that determined cortisol on samples not related to the agri-food sector. After identifying and screening, 21 publications were selected to assess the importance of cortisol and its measurement in the food industry.

## 3. Revealing Cortisol in the Agri-Food Sector

According to the search strategy, the publications retrieved evidenced that in the last 11 years, the research carried out on the agri-food industry has focused on the natural occurrence of cortisol in food derivatives and the effect of environmental stressors. Additionally, some researchers have been working on optimizing sample conservation and handling techniques and validating analytical procedures for clean-up to overcome low cortisol concentrations. Lastly, monitoring cortisol to identify prohibited substances in farm animals intended for consumption has also been studied. Next, we review and discuss the findings of these studies, grouping the research carried out in the livestock and fish farming sectors.

### 3.1. Impact of Monitoring Cortisol on the Agri-Food Sector

#### 3.1.1. Livestock Animals

Cortisol monitoring in cattle represents a challenge to the food industry due to (i) the diversity of matrices that can be analyzed, (ii) the sampling collection ranging from non-invasive to invasive methods, (iii) cortisol levels being very low, and (iv) the requirement for a suitable quantification method.

According to our search, the non-invasive matrices employed in the last ten years to assess cortisol levels in cattle and swine were saliva, hair, and urine. In 2015, Rey-Salgueiro et al. [17] determined the swine’s baseline or non-stress level of salivary cortisol according to their circadian rhythm. The analysis of 48 saliva samples revealed that a cortisol content of 3.0 μg/L can be considered a biomarker to indicate the maxima non-stress levels in different pig breeds at farms. In another study, Binz et al. [18] quantified the corticosteroid by developing and validating an LC-MS/MS method that used the surrogate analyte ^13^C- labeled cortisol, having found a content of 1.1 pg/mg in cow hair. On the other hand, Pavlovic et al. [19] measured cortisol levels in urine samples from cows and bullocks. Results showed that in cows its concentration ranged from 2.5 to 5.0 ppb, whereas in bullocks it was higher, from 2.5 to 12.9 ppb, a difference that authors associated with the fact that younger and male animals could be more affected by stress.

The natural occurrence of cortisol in food from animal origins has also been monitored since this hormone is considered an endocrine active substance (EAS) that can interact or interfere with normal hormone action. One of the vital sources of this substance for humans is milk. The cortisol content in 103 milk samples from Swiss Holstein cow milk and the difference in the number of lactations (number of calvings) on hormone content in milk were assessed for the first time by Goyon et al. [11] after the development and validation of an LC-MS/MS method. Results showed that cortisol was present in all samples, varying from 37 to 1466 ng/kg, and that no significant differences among milk from cows with different lactation numbers were evidenced. Milk intake with this glucocorticoid can negatively affect human consumers and rearing livestock. In another study, Chiesa et al. [20] found an average cortisol content of 2.56 ng/mL in milk replacers used as dairy feed replacement in calf rearing. On the other hand, it has been reported that intrafollicular cortisol levels may directly impact dairy cows’ cumulus cell lipolysis and oocyte quality. The analysis of follicular fluid from dairy cows showed the presence of cortisol ranging between 5.7 and 18.2 ng/mL [21].

However, determining the hormone levels in animal tissues or their excretion samples can only be accomplished if the sample handling and conservation are adequate for each case. In terms of sample protection, De Clercq et al. [13] defined suitable conditions for sample handling and storage through the development of an ultra-high performance liquid chromatography–high-resolution mass spectrometry (UHPLC–HRMS) technique to investigate the stability of glucocorticoids in bovine urine under various storage temperatures [−80 °C, −20 °C, 4 °C, or at room temperature (15–20 °C)] for up to 20 weeks. The authors recommended filter-sterilizing the urine and preserving it under acidic conditions (preferentially at pH 3 at −80 °C, or at least −20 °C) to preserve the glucocorticoids in bovine urine for up to 20 weeks. Employing the same experimental design, the authors then assessed cortisol stability in cow feces. For this type of sample, it was concluded that (i) freezing (−80 °C) fecal samples without any chemical treatment, such as the addition of ethanol, increased cortisol recovery, (ii) lyophilization of the feces is a good alternative for long-term storage, and (iii) the removal of water from the matrix prolonged storage up to 10 weeks at room temperature without significant (*p* < 0.05) loss of the glucocorticoid [15].

Nevertheless, since cortisol levels are extremely low in most cases, it is necessary to implement a sample pre-treatment before analytical analysis. To overcome the limitations of urine samples related to extremely low cortisol levels, Chiesa et al. [22] developed and validated a simple and unique immunoaffinity clean-up procedure, which was applied to 20 bovine bile samples (invasive sampling), followed by LC-ES-MS-MS. The analysis revealed that cortisol content ranged from 0.3 to 6.8 ng/mL with an average of 2.3 ng/mL. In a later study, the same authors decided to compare two matrices, urine and bile (from male veal calves, young bulls, and dairy cows), to understand whether the detection of steroids in the latter was more feasible. The authors found that cortisol, cortisone, pseudoendogenous prednisolone, and prednisone are present at higher concentrations, up to seven times higher, in the urine. On the other hand, dexamethasone was found in 10 out of 53 bovine bile samples, but only in one urine sample [23].

As previously mentioned, cortisol is a hormone involved in critical behavioral and metabolic processes of living organisms, such as inflammation, immune function, stress response, and reproduction. The discovery of those properties, particularly the anti-inflammatory action, leads to the production of synthetic glucocorticoids that, besides their greater anti-inflammatory properties, also induce body weight gain in livestock animals. The most applied glucocorticoids as therapeutic drugs in veterinary medicine are betamethasone, dexamethasone, methylprednisolone, and prednisolone. In recent years, cortisol monitoring in the food sector has increased since it allows the identification of the administration of those illicit anabolic substances (anabolic hormones and synthetic corticosteroids) that can represent a potential risk to consumers’ health [13,19]. It has been reported that the simultaneous determination of urinary cortisol and cortisone, together with prednisolone, prednisone, and at least 20β-dihydroprednisolone among metabolites, is likely to represent a more practical approach for the correct assessment of therapeutic administration of prednisolone on bulls and cows [12].

#### 3.1.2. Aquatic Species

In aquatic species, shifts in hormone status are more prone to occur due to environmental stressors or human actions than due to the presence of contaminants or pollutants in water, handling, and transportation. The rising cortisol levels in fish due to stress can lead to disease, alterations in growth rate and reproduction cycle, and in some cases death. Thus, it is essential to monitor or assess the variations in cortisol levels in wild marine species or those produced in aquaculture facilities to protect them from environmental changes, guarantee their normal development, ensure their survival and obtain the best quality food products.

Fish production in aquaculture facilities is more susceptible to a variable number of stressors than in natural environments. Producing juvenile fish (larviculture), known as larvae, is a critical step in this industry since it has a low and unpredictable survival rate and a high disease susceptibility. Implementing probiotics in this stage of the aquaculture cycle positively impacts fish welfare by alleviating the general stress response. For example, administering the probiotic *Vibrio lentus* to sea bass larvae reduced the concentration of the glucocorticoid profile, significantly attenuating cortisol levels from 6.99 to 1.41 μg/kg [24].

Additionally, monitoring the stress level of female fishes is pivotal to avoiding disturbances in the reproductive cycle, egg size, gamete quality, and fecundity, which are critical for the environment and food industry. Bussy et al. [9] demonstrated that the stress that farmed female sturgeon experience is transmitted to the eggs (caviar). After optimizing an extraction procedure and LC-MS/MS methods, authors verified that non-fertilized lake sturgeon (*Acipenser fulvescens*) eggs had significantly higher cortisol content than fertilized eggs, 543.4 ± 194.4 pg/g and 39.2 ± 11.7 pg/g, respectively.

Another concern for the fish farming industry is water pollution. This problem could lead to significant environmental changes for fish, resulting in a disease or, in the worst-case scenario, a mass death. For these cases, monitoring cortisol levels in fish indicates stressful conditions. In 2017, a research group analyzed the plasma cortisol levels in *Oreochromis* sp. to investigate the effect of cadmium pollution on their endocrine stress response. Firstly, the LC_50_ value (19.6 mg/L) was calculated, and the fishes were exposed to waterborne cadmium at 0%, 2%, 2.65%, 3.3%, 5%, and 10% of 96 h LC50 or to long-term exposure (20 days) to cadmium. The results showed that the fish endocrine system was exhausted, which means that cortisol levels decreased between 85 and 91% when exposed to 1.0–2.0 mg/L and a 52–78% suppression of cortisol release under 0.4–0.66 mg/L in a long-term exposure assay [25]. In another study, plasma cortisol levels were about 9.5 times higher in female fish collected from a lake contaminated with organochlorine pesticides from farming activities than in females collected from a reference lake with no contaminant input [26].

Moreover, Li et al. [27] demonstrated that small changes in an aquatic environment could impair cortisol content in zebrafish, depending on fish gender. After the acute exposure of zebrafish to 300 mg/mL of caffeine or 1% ethanol for 5 min, cortisol levels of male zebrafish were reduced to around 19 ng/g and 16 ng/g, respectively, compared to the control group (23 ng/g). In the female group, the cortisol level in the caffeine treatment increased significantly, from 13.3 ng/g up to 25 ng/g. Moreover, in another study, the analysis of the whole tissue homogenate of zebrafish revealed that cortisol levels were significantly lower, up to four times lower, approximately, in female zebrafish compared to males [14]. In another study, Atlantic salmon released from aquaculture facilities into seawater cages experienced this as a stressful event. Still, cortisol levels decreased significantly, varying from 437 ± 293 ng/g after four days to less than 80 ng/g after eight days at sea [28].

In aquatic toxicology, monitoring steroids is vital for detecting endocrine-disrupting compounds (EDCs) in water. EDCs affect the reproductivity and development of vertebrates and influence normal steroid production activity. The analysis of blood plasma from female fathead minnows exposed for 48 h to waterborne fadrozole (EDC substance) enabled the detection of cortisol in these fish for the first time. Additionally, the authors conducted a second experiment that exposed spawning Japanese medaka to waterborne fadrozole (0, 2, 10, and 30 µg/L) for 21 days. The analysis of those water samples did not show any correlation between cortisol and EDC exposure; however, it was found through the analysis of control Lake Superior water samples that cortisol was present in the water in its free form [10].

On the other hand, even the simple act of handling for a reduced period rapidly induces stress on fish, leading to an exponential production of cortisol, as demonstrated by Wish et al. [29]. In their experiment, rainbow trout were vigorously chased with a net, caught, briefly lifted out of the water and then released over a total period of 5 min and corralled in a smaller area of the tank. The authors observed that cortisol was significantly induced after the acute stress exposure after 4 and 48 h in both brain and liver fish tissues. Cortisol reached maximum content after 4 h and significantly decreased around 1.5 times after 48 h in both tissue samples. Additionally, in fish brain extracts the highest content was 0.99 ± 0.26 ng/g 4 h post-stress, and in liver extracts its concentration ranged from 0.05 ± 0.02 ng/g to 0.17 ± 0.03 ng/g, there also being a significant difference between control and stress exposure at both sampling times. Moreover, fish bile has been reported as an optimal matrix to measure stress levels, since cortisol metabolites present in the bile are not affected by the stress induced during sample collection [30].

According to the papers reviewed and discussed above, it can be concluded that in the marine food industry or research, the traceability of cortisol, along with its direct impact on a living organism in several growth stages or overall welfare, seems to be well understood and widely explored compared to investigations conducted in livestock animals.

### 3.2. Analytical Methods

Over the years, several methods have been applied to identify and quantify the cortisol levels in agri-food samples, including radioimmunoassay (RIA), enzyme-linked immunosorbent assay (ELISA), gas chromatography (GC), and liquid chromatography coupled to mass spectrometry (LC-MS). According to the search strategy, this section reviews the extraction or pre-concentration techniques commonly applied to agri-food samples and the advantages of performing the analysis using LC-MS methods.

#### 3.2.1. Techniques to Extract Cortisol on Agri-Food Samples

Cortisol measurements in any agri-food matrix require a proper collection and storage method to avoid steroid degradation and sample contamination. Additionally, since this analyte is found in low concentrations, its measurement often requires an extraction or pre-concentration step that is typically achieved using liquid–liquid extraction (LLE) or solid-phase extraction (SPE), as detailed in Table 1.

**Figure 2:**
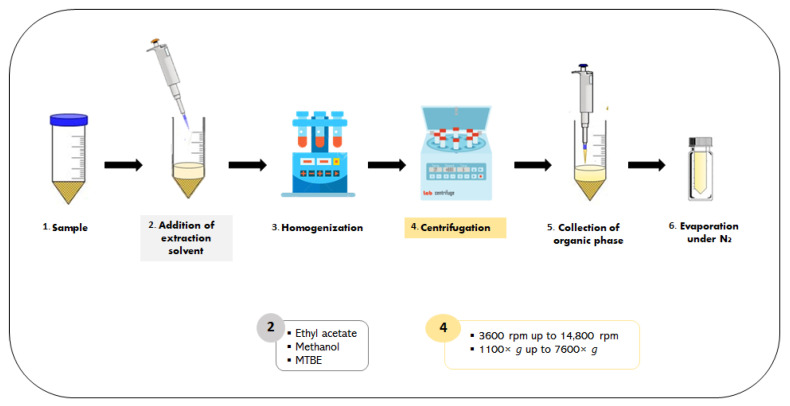
Schematic representation of the liquid–liquid extraction of cortisol with commonly applied extraction solvents and a range of centrifugation conditions.

LLE is a routine sample pre-treatment applied before an analytical process that, like any other technique, has advantages and disadvantages. LLE is a versatile procedure since it can increase or enhance selectivity by removing matrix-interfering species from the target analyte or concentrating the analyte from a large sample volume [31]. On the other hand, implementing this sample treatment is not environmentally friendly and is potentially harmful to the operator, because it requires large amounts of toxic or flammable chemicals, resulting in a time-consuming, expensive and labor-intensive operation [31]. Briefly, this procedure involves mixing the sample with an extraction solvent, and then the mixture is centrifugated and the resulting extract is generally evaporated under nitrogen gas. A schematic of the analytical method is presented in Figure 2. For agri-food samples, the most common extraction solvents used are methyl tertiary-butyl ether (MTBE), methanol and ethyl acetate [13,14,15,17,18,26,28].

**Figure 3:**
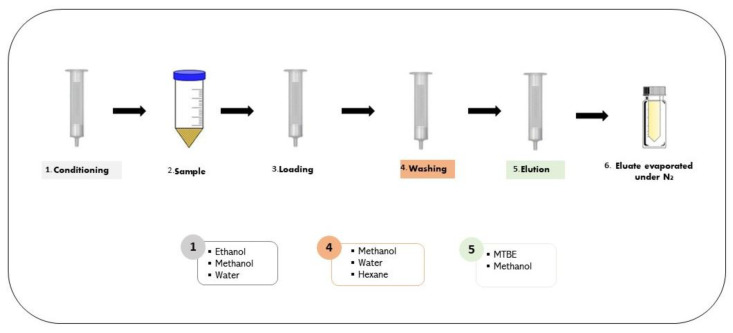
Schematic representation of the solid–liquid extraction of cortisol with the indication of the commonly applied solvents.

SPE methods have been widely applied to overcome the drawbacks of LLE and implement greener solutions for sample preparation (Table 1). Compared with LLE, SPE is a more efficient process since it results in a higher recovery of the target analyte by using a reduced volume of chemicals and does not require a phase separation. However, this method has a higher cost per sample, requires several steps, has a wide range of chemical manipulation and pH conditions, and is necessary to learn how to perform the method [32,33]. In summary, to perform an SPE, it is necessary to condition the column/cartridge, load the sample into the cartridge, and wash and elute the target compounds with a suitable extraction solvent. The resulting extract is evaporated under nitrogen gas. A scheme of SPE steps and the most common solvents used are presented in Figure 3. In food science research, the most applied cartridges are the ones with a reversed-phase sorbent, generally activated by water, methanol and ethanol, either loading a single solvent or a mixture, as presented in Table 1.

#### 3.2.2. Liquid Chromatography

LC-MS is a powerful tool used to determine cortisol levels in food samples due to its high sensitivity, selectivity, specificity, and automation, allowing the analysis of multiple steroids without the need for a previous hydrolysis or derivatization step. The application of LC-MS or tandem mass spectrometry (MS-MS) is the technique of choice for measuring steroid hormones, as has been reported [12,17,18,22,23]. Additionally, some studies reported the utilization of MS with electrospray ionization (ESI), atmospheric pressure photoionization (APPI), time-of-flight (TOF) mass spectrometry [10,19,27], or high-performance liquid chromatography (HPLC) [25,30]. The variations between the applied LC-MS systems are correlated with the steroid nature [21]. In general, the concentration range of cortisol varies from 0.003 to 2720 ng/mL in samples from the agri-food sector (Table 1). The reported values obtained for the limit of detection (LOD) ranged between 0.04 to 1.11 ng/mL, whereas the limit of quantification (LOQ) varied from 0.025 to 3.70 ng/mL [14,28,29,34], as presented in Table 1.

## 4. Added Value

This systematic review discussed, for the first time, the impact of cortisol on the agri-food sector and the analytical methods applied to measure the analyte. Most studies have focused on marine species, particularly aquaculture production, revealing that cortisol monitoring is a valuable tool to control and evaluate fish welfare, contributing to the development of intensive aquaculture. Regarding cattle, the scenario is different since the studies available are associated more with sample preparation and cortisol quantification than the drawbacks that cortisol could have on those animals. Additionally, the available data demonstrate that obtaining a sample to measure the analyte is frequently invasive for the species, and its handling is laborious. Even so, additional studies are necessary to clarify the impact of cortisol on living organisms continuously and to develop new technologies or strategies to fill this research gap and help the food industry to ensure food quality and security and continuous sustainable growth.

## 5. Future Trends, Perspectives, and Technologies

The exponential growth of the world’s population demands that the agri-food sector ensures the sustained production and commercialization of foods that meet all nutrition, food security, and quality requirements.

Although monitoring cortisol levels is an excellent tool for early warning signals, in most cases it is an invasive process that results in the loss of animals intended for consumption. Implementing new technologies and artificial intelligence will allow the real-time tracking of cortisol levels and other biomarkers without harming living beings. For example, developing a biosensor system for aquaculture tanks to monitor biomarkers or detect the presence of microplastics directly from the water in the tank could be a great tool to ensure fish welfare and develop intensive aquaculture [34]. On the other hand, further investigations and innovation for monitoring cortisol levels in livestock animals are critical to enhance farming and ensure food quality and security for consumption. Some studies are in progress in the framework of the DigiAqua project [34], where advances have been made to achieve a cortisol biosensor that can be installed in fish tanks [35].

## 6. Conclusions

A review of the scientific literature revealed that the monitorization of cortisol in the agri-food sector in the past eleven years (2012–2022) has been limited, particularly in the livestock sector. According to the results, the fish farming sector has utilized knowledge about the advantages and weaknesses of cortisol as a stress hormone in fish. Cortisol monitoring has facilitated solid industry development at all production phases (juveniles to adults) to the final product. In contrast, little is known regarding the possible impact cortisol levels could have on the reproductive cycle and development of cattle, or the quality of the food derivatives and the safety of the consumers due to this hormone’s endocrine action. Indeed, further in-depth studies are needed in the livestock sector.

According to this review, LC-MS detects cortisol with high sensitivity, selectivity, and specificity, but relying on this technique has some drawbacks, not only due to the need to implement extraction or pre-concentration methods before the analytical analysis, but also due to the higher costs associated with it, and sampling invasiveness. Developing technologies incorporating sensing devices may yield a novel, fast, real-time, reliable, and non-invasive way for the agri-food sector to monitor cortisol levels, consequently enhancing animal welfare and sustainable development in the agri-food industry.

## Figures and Tables

**Table 1:** Review of methodologies applied to cortisol extraction on agri-food samples followed by LC analysis.

Species [Ref.]	Matrix	Extraction Technique	Extraction Solvent	Type of Cartridge	Kind of Sorbent	Conditioned Solvent	Washing Solvents	Centrifugation Conditions	Chromatographic Technique	Concentration Range	LOD	LOQ
Bovine [19]	Urine	SPE	Methanol	Oasis HLB	Reversed-phase	Methanol, water	10% methanol, 2% ammonia, 50% methanol		LC-ESI-MS	2.8–5.0/2.5–12.9 μg/L	N.A.	2.5 μg/L
Bovine [13]	Urine	LLE	MTBE					5500× *g*, 5 min, 7 °C	U-HPLC	0.25 to 10 μg/L	0.10–0.25 µg/L	0.30–0.83 µg/L
Bovine [22]	Bile	Immunoaffinity columns (IAC)	Ethanol:water (70:30 *v*/*v*)		N.A.	Ethanol:water (70:30 *v*/*v*)	Wash buffer, water		LC-MS	0.3–6.8 ng/mL	N.A.	N.A.
Cow [15]	Feces	LLE and SPE	(MTBE	Isolute C18 (EC) (500 mg, 10 mL)	Reversed-phase	Methanol, water	Water, methanol:water (20:80), n-hexane	7600× *g*, 10 min, 7 °C	U-HPLC-MS	1.25–50 μg/kg	0.55 µg/L	0.70 µg/L
Bulls and cows [12]	Urine	LLE	Diethylether					N.A.	LC-MS/MS		0.1 ng/mL	N.A.
	Cows									<LOQ.		
	Bulls									0.63–3.44 ng/mL		
Cod [30]	Bile	SPE	Methanol	SPEC, 820 mg	Reversed-phase	Methanol, deionized water	Deionized water		HPLC	N.A.	N.A.	N.A.
Bulls, cows, and veal calves [23]	Bile	Immunoaffinity columns (IAC)	Ethanol:water (70:30, *v*/*v*)		N.A.	Ethanol:water; 70:30, *v*/*v*	Wash buffer, water		LC-MS/MS	2.40 ± 1.86 ng/mL (calves); 3.50 ± 1.64 ng/mL (bulls); 5.94 ± 9.28 ng/mL (cows)	N.A.	N.A.
	Male veal calves											
	Young bulls											
	Cows											
	Urine									17.9 ± 14.7 ng/mL (calves); 14.4 ± 11.8 ng/mL (bulls); 22.0 ± 17.5 ng/mL (cows)		
	Male veal calves											
	Young bulls											
	Cows											
Cows [21]	Follicular fluids	LLE	N.A.					15 min at 14,000 rpm at 4 °C	LC-MS/MS	analytical 1.0–499.0 ng/mL	N.A.	2.5 ng/mL
Pig [17]	Saliva	LLE and SPE	Ethyl acetate:Ethyl ether (1:1)	Octadecyl-carbon (C18)	Reversed-phase	Methanol, water	Water, water:acetone (4:1), hexane	5 min at 3500× *g*	LC-MS/MS	0.4–10 µg/L	0.02 µg/L	0.05 µg/L
				Silica		Hexane, ethyl acetate						
Cow [18]	Hair	LLE	Methanol					16 h in an ultrasonic bath at 55 °C	LC-MS/MS	1–500 pg/mg	0.2 pg/mg	1 pg/mg
Calf [20]	Powdered milk	LLE and IAC	n-Hexane (LLE) and Ethanol:water (70:30 *v*/*v*) (IAC)	N.A.	N.A.	Ethanol:water (70:30 *v*/*v*)	Wash buffer, water	2500× *g*	LC-MS/MS	0.76–3.81 ng/mL	N.A.	N.A.
Bovine [11]	Milk	LLE and SPE	Methanol (LLE)	Supelclean ENVI-Carb (500 mg, 6 mL)	Reversed-phase	Dichloromethane, methanol, water	Methanol	4500× *g* at 4 °C for 10 min	LC-MS/MS	37–1466 ng/kg	N.A.	N.A.
			Dichloromethane:methanol (7/3, *v*/*v*)	Sep-Pak amino-propyl (500 mg, 6 mL)	Reversed-phase	Dichloromethane:methanol (7/3, *v*/*v*)	N.A.					
Lake sturgeon [9]	Eggs	LLE	Ethyl acetate					9000× *g*, 10 min, 4 °C	LC-MS/MS	0.25–100 ng/mL	0.025 ng/mL	0.1 ng/mL
Fish (*Oreochromis* sp.) [25]	Blood plasma	LLE	Dichloromethane					3600 rpm for 15 min	RP-HPLC/UV	50–250 ng/mL	0.87 ng/mL	2.90 ng/mL
								3600 rpm for 5 min				
Fish [24]	Sea bass larvae	LLE and SPE	Methanol (LLE) and Water:methanol (20:80; *v*/*v*) (SPE)	Grace Pure™ SPE C18-Max (500 mg, 6 mL)	Reversed-phase	Methanol, water	Water/methanol (65:35; *v*/*v*)	3500× *g* at 7 °C for 10 min	UPLC-MS/MS	1.388–50.000 µg/kg	N.A.	N.A.
Fish [14]	Plasma and tissue homogenates of fish	LLE	MTBE					1100× *g* for 10 min at 4 °C	UPLC-MS/MS	0.003–200 ng/mL	10 µL sample: 0.5 ng/mL	10 µL sample: 0.025 ng/mL
Fish [10]	Fathead minnow plasma	SLE	Dichloromethane	Phenomenex Novum, 1 cc		N.A.	Hexane		LC-APPI-MS/MS	0.01–10 ng/mL	0.6 ng/mL	1.0 ng/mL
	Japanese medaka exposure to water	SPE	90:10 ethyl acetate:MeOH (*v*/*v*) 2% NH_4_OH in MeOH	Strata-X, 200 mg	Reversed-phase	Ethyl acetate, methanol, and water	93:5:2 water:MeOH:acetic acid (*v*/*v*/*v*) and 93:5:2 water:MeOH:ammonium hydroxide (*v*/*v*/*v*)			0.1–100 ng/mL	N.D.	0.5 ng/L
Fish (Largemouth Bass (*Micropterus salmoides*) [26]	Plasma	LLE	MTBE					5200× *g* for 10 min	LC-MS/MS	0.05–200 pg/μL	N.A.	0.05 pg/µL
Fish (Zebrafish - *Danio rerio*) [27]	Tissue homogenates	SPE	75% methanol/water containing 2% acetic acid	Oasis HLB Vac	Reversed-phase	Methanol, water	30% methanol/water containing 2% acetic acid		UPLC-TOF-MS	0.3–200 ng/mL	0.1 ng/mL	0.3 ng/mL
Fish (Atlantic salmon) [28]	Feces	LLE	MTBE, NaCl					2000× *g* for 5 min	LC-MS/MS	0–100 ng/mL	0.04 ng/mL	0.09 ng/mL
Fish (rainbow trout) [29]	Liver and brain	Liquid-solid extraction	Cold 35:65 Milli-Q water:acetonitrile (4 °C, pH 7)					14,800 rpm for 15 min	LC-MS/MS	1.0–1000 pg/μL	1.11 pg/μL	3.7 pg/μL

N.A.—information not available; N.D.—information not determined.
